# Utility of near‐surface phenology in estimating productivity and evapotranspiration across diverse ecosystems

**DOI:** 10.1002/jeq2.70043

**Published:** 2025-06-02

**Authors:** Sander O. Denham, Dawn M. Browning, Adam P. Schreiner‐McGraw, Russell L. Scott, Brent Dalzell, Gerald N. Flerchinger, Patrick E. Clark, Sarah Goslee, David L. Hoover, Marcy Litvak, Marguerite Maritz, David Huggins, Claire L. Phillips, John Prueger, Joe Alfieri, Rosvel Bracho, Maria Silveira, Craig W. Whippo

**Affiliations:** ^1^ USDA‐ARS Jornada Experimental Range Las Cruces New Mexico USA; ^2^ USDA‐ARS Cropping Systems and Water Quality Research Unit Columbia Missouri USA; ^3^ USDA‐ARS Southwest Watershed Research Center Tucson Arizona USA; ^4^ USDA‐ARS Soil and Water Management Research Unit St. Paul Minnesota USA; ^5^ USDA‐ARS Northwest Watershed Research Center Boise Idaho USA; ^6^ USDA‐ARS Pasture Systems and Watershed Management Research Unit University Park Pennsylvania USA; ^7^ USDA‐ARS Rangeland Resources and Systems Research Unit Fort Collins Colorado USA; ^8^ Department of Biology University of New Mexico Albuquerque New Mexico USA; ^9^ Biological Sciences University of Texas at El Paso El Paso Texas USA; ^10^ USDA‐ARS Northwest Sustainable Agroecosystems Research Unit Pullman Washington USA; ^11^ USDA‐ARS National Laboratory for Agriculture and the Environment (NLAE) Ames Iowa USA; ^12^ USDA‐ARS Hydrology & Remote Sensing Lab Beltsville Maryland USA; ^13^ School of Forest, Fisheries, & Geomatics Sciences University of Florida Gainesville Florida USA; ^14^ USDA‐ARS Northern Great Plains Research Laboratory Mandan North Dakota USA

## Abstract

Agroecosystems, which include row crops, pasture, and grass and shrub grazing lands, are sensitive to changes in management, weather, and genetics. To better understand how these systems are responding to changes, we need to improve monitoring and modeling carbon and water dynamics. Vegetation Indices (VIs) are commonly used to estimate gross primary productivity (GPP) and evapotranspiration (ET), but these empirical relationships are often location and crop specific. There is a need to evaluate if VIs can be effective and, more general, predictors of ecosystem processes through time and across different agroecosystems. Near‐surface photographic (red‐green‐blue) images from PhenoCam can be used to calculate the VI green chromatic coordinate (G_CC_) and offer a pathway to improve understanding of field‐scale relationships between VIs and GPP and ET. We synthesized observations spanning 76 site‐years across 15 agroecosystem sites with PhenoCam G_CC_ and GPP or ET estimates from eddy covariance (EC) to quantify interannual variability (IAV) in the relationship between GPP and ET and G_CC_ across. We uncovered a high degree of variability in the strength and slopes of the G_CC_ ∼ GPP and ET relationships (*R* ^2^ = 0.1 ‐ 0.9) within and across production systems. Overall, G_CC_ is a better predictor of GPP than ET (*R* ^2 ^= 0.64 and 0.54, respectively), performing best in croplands (*R* ^2 ^= 0.91). Shrub‐dominated systems exhibit the lowest predictive power of G_CC_ for GPP and ET but have less IAV in slope. We propose that PhenoCam estimates of G_CC_ could provide an alternative approach for predictions of ecosystem processes.

## Core Ideas

- The ability of green chromatic coordinate (G_CC_) to predict production (gross primary productivity [GPP]) or evaporative losses (ET) is highly variable within and across agroecosystems.
- G_CC_ is a better predictor of GPP than of ET.
- G_CC_ as a predictor of GPP or ET is less temporally variable in grasslands than in agriculture or shrublands.

**Abbreviations**
EC: eddy covariance
ET: evapotranspiration
G_CC_
: green chromatic coordinate
GPP
: gross primary productivity
IAV: interannual variability
LTAR: Long‐Term Agroecosystem Research
NDVI: normalized difference vegetation index
PFT: plant functional type
SCVTG: Seasonal Characteristics of Vegetation Types and Growth
VI: vegetation index

## 1 INTRODUCTION

Agroecosystems are consistently subject to changes in management, vegetation communities, and weather. For example, the spread of invasive species and frequency and severity of drought are increasing (Bradley, 2009; Bradley et al., 2009; Nagler et al., 2005; Nagy et al., 2021). These invasions can promote more frequent fires and alter the carbon storage in aboveground biomass (Stark & Norton, 2015). In some regions (i.e., southwest US), woody shrub encroachment is increasing and is not likely to slow as challenges associated with novel climate conditions become more prominent (Schreiner‐McGraw et al., 2020). In croplands, increased interest in cover cropping can have mixed impacts, both (1) avoiding periods of bare soil associated with greater risk of erosion and nitrogen leaching losses (Battany & Grismer, 2000) and (2) yield losses due to resource competition (Qin et al., 2021). To better understand how both natural and cropping systems are responding to environmental changes, we need to improve monitoring and modeling carbon and water dynamics.

Over the past several decades, research into plant carbon uptake and water budgets has been critical to our understanding of trends in ecosystem productivity and the effects of drought frequency and severity (Jiao et al., 2021). Ecosystem photosynthesis, or gross primary productivity (GPP), is determined largely by long‐term responses of vegetation to environmental conditions that control the quantity of foliage and thus the amount of light that is captured and absorbed. Eddy covariance (EC) flux towers directly monitor GPP and are increasingly used for continuous measurement of carbon and water fluxes. However, EC systems are expensive to deploy, require regular maintenance to ensure data quality, and are not considered practical for many research programs. Vegetation indices (VIs), in contrast, tend to represent photosynthetic capacity rather than actual photosynthesis of an ecosystem (Zhu et al., 2023). Canopy greenness from VIs generally increases as leaf area increases, though a saturation effect emerges when canopies become denser with vegetation (Liu et al., 2012). Efforts in advancing our ability to model GPP and evapotranspiration (ET) have included regression models that incorporate VIs such as normalized difference vegetation index (NDVI; Del Grosso et al., 2018) and have also included parametric models, which assume a known functional form (e.g., light‐use efficiency, Medlyn, 1998), process‐based models using mechanistic details (Ivanov et al., 2008), and machine learning algorithms (Menefee et al., 2023). For regression‐based models, VIs are commonly used as an important explanatory variable for satellite‐derived estimates of GPP along with other meteorological drivers of productivity (i.e., temperature, rainfall) (Hufkens et al., 2016; Post et al., 2022). While many of these models are site specific and have not been tested over a broader spatial domain, both GPP and ET have been predicted with a reasonable degree of success by using NDVI combined with ground meteorological data to map regional carbon fluxes (Wylie et al., 2003) and riparian zone ET (Nagler et al., 2005). However, near‐surface imagery provided by digital cameras at the field scale has become more ubiquitous across many landscapes and may provide a more accurate representation of rapid, short‐term vegetation changes caused by stochastic events (e.g., heat waves) to better inform GPP and ET estimates in some land cover types.

The VI, green chromatic coordinate (G_CC_), can be derived from PhenoCam images to quantify canopy greenness, which is calculated by dividing the green spectral band by the sum of red, blue, and green spectral bands (Equation 1). G_CC_ has proven to be highly correlated with GPP in wetlands (Knox et al., 2017), forests, and grasslands (Toomey et al., 2015). The relationship between G_CC_ and GPP tends to be strongly linear at lower values of G_CC_ and weakens at higher G_CC_ in deciduous broadleaf forests (Toomey et al., 2015). However, to our knowledge, year to year variability in the strength of the relationships of G_CC_ and GPP or ET has not been evaluated across agroecosystems. Continuing with these efforts to further develop cost‐effective and widely applicable productivity and ET models is valuable for increasing our understanding of how carbon and water cycling respond to land‐use and land‐cover change.

Long‐term research initiatives, such as USDA's Long‐Term Agroecosystem Research (LTAR) network, and the Long‐Term Ecological Research (LTER), AmeriFlux, and PhenoCam networks are vital resources that allow us to capture and better understand environmental change and its impacts. The PhenoCam Network archives sub‐daily time‐series imagery documenting vegetation phenology of North America, providing a unique resource for understanding relatively short‐term land cover changes (Richardson, 2023). The LTAR network was established in 2014 to address challenges associated with agricultural sustainability and includes 19 sites distributed across the United States representing diverse agroecosystems (Kleinman et al., 2018). Many LTAR sites have deployed both PhenoCams and EC flux towers as part of their ongoing research capable of capturing real‐time responses to coordinated experimental treatments (Browning et al., 2021; Spiegal et al., 2018) and provide an opportunity to leverage long‐term, automated data streams to evaluate biosphere‐atmosphere interactions across multiple vegetation types and agroecosystems.

**Core Ideas**

- The ability of green chromatic coordinate (G_CC_) to predict production (gross primary productivity [GPP]) or evaporative losses (ET) is highly variable within and across agroecosystems.
- G_CC_ is a better predictor of GPP than of ET.
- G_CC_ as a predictor of GPP or ET is less temporally variable in grasslands than in agriculture or shrublands.

We combine two near‐surface, high temporal frequency data streams to explore the relationships between near surface VI (G_CC_) and observations of carbon and water fluxes to evaluate the ability of PhenoCam imagery to estimate ecosystem productivity and ET. We explore patterns of vegetation greenness from PhenoCams and eddy‐flux‐derived ecosystem‐level GPP and ET across space (i.e., diverse agroecosystems) and time to determine the sensitivity of daily GPP or ET to changes in G_CC_. Our objectives are (1) to assess the interannual variability (IAV) in the sensitivity of both GPP and ET to incremental changes in G_CC_ and (2) to evaluate the consistency and strength of the predictive power of G_CC_ to both GPP and ET across diverse agroecosystems within the continental United States. We hypothesize that GPP and ET as a function of G_CC_ will be predominantly linear as productivity increases with increasing leaf area, allowing for greater productivity and larger surface for water to transpire. We expect, however, some degree of nonlinearity once peak greenness is achieved, and productivity and water use are more reliant on day‐to‐day meteorological drivers rather than leaf area (Toomey et al., 2015). We expect that these relationships will be stronger in cropland (agriculture) and grazing (grasslands) systems due to the more homogenous land cover compared to the heterogenous vegetation cover of grazing shrublands (Browning et al., 2021). We also expect that G_CC_ will be a better predictor of GPP than ET due to the biological nature of G_CC_ in representing leaf area (whereas ET incorporates both vegetative water loss and evaporation from soil) and that the year‐to‐year variability of GPP to G_CC_ will be greater in cropland systems due to active management aimed at specific objectives. Our focus was on when and where vegetation greenness is coupled with ecosystem land‐atmosphere carbon and water fluxes across various vegetation (i.e., agriculture, shrublands, and grasslands) and production types (i.e., grazing and cropland) across the United States.

## 2 METHODS

### 2.1 Site descriptions

**FIGURE 1:**
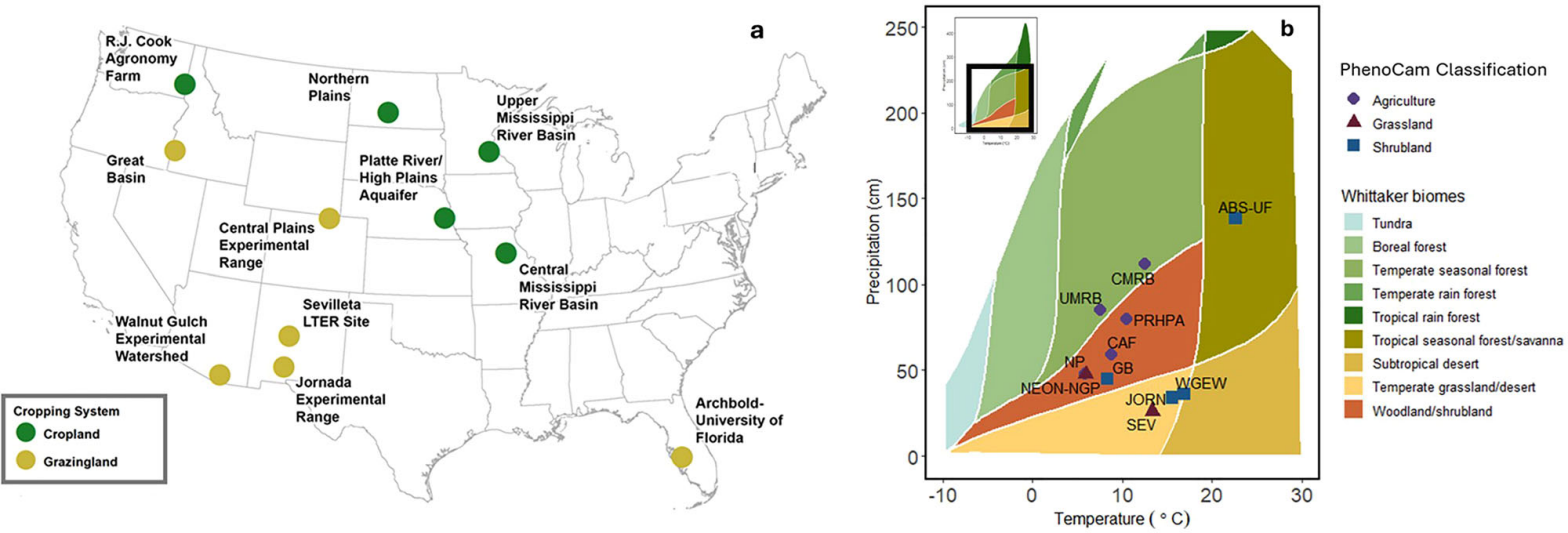
Map of site locations: (a) Sites situated in climate space are represented here as mean annual temperature and mean annual precipitation in the context of Whittaker biome designation. Inset panel shows full extent of Whittaker biome plot with box roughly around zoomed in area (b), figure modified using plotbiomes R package (Ștefan & Levin, 2018). Site IDs are referenced as the Long‐Term Agroecosystem Research (LTAR) or Long‐Term Ecological Research (LTER) code, see Table 1 for Ameriflux and PhenoCam site ID.

We selected LTAR network sites with co‐located EC towers and PhenoCam imagery in diverse agroecosystems across the coterminous United States representing rangelands (either shrublands or grasslands) and agriculture (a mix of cropland and pastures; Table 1; Figure 1). We used a 5‐year window (2016–2021) of overlapping PhenoCam and EC flux tower data representing the greatest site overlap of available data. Sites are differentiated both by their agroecosystem production type (e.g., cropland or grazing; Speigal et al., 2018) as well as by their vegetation designation on the PhenoCam network (e.g., agriculture, grassland, or shrubland; Table 1; Richardson, 2023). We include the Sevilleta LTER network shrubland (US‐Ses) and grassland (US‐Seg) sites to increase sample size of grass and shrublands.

**TABLE 1:** Details for PhenoCam and eddy covariance towers used in this study along with details of the eddy covariance data sourcing and processing.

Location LTAR code	EC tower	PhenoCam name	Production type	PhenoCam vegetation ROI	EC reference	Data range	# Years used
CAF	US‐CF2	cafcookeastltar01	Cropland	Agriculture	Russell et al. (2019)	2017–2020	4
CAF	US‐CF3	cafcookwestltar01	Cropland	Agriculture	Russell et al. (2019)	2017–2021	5
JORN	US‐Jo1	jerbajada	Grazing	Shrubland		2016–2020	5
CMRB	US‐Mo1	goodwater	Cropland	Agriculture	Schreiner‐McGraw et al. (2023)	2016–2021	6
PRHPA	US‐Ne1	mead1	Cropland	Agriculture	Suyker et al. (2005	2016–2020	5
NP	US‐NP1	mandanh5	Cropland	Agriculture	Saliendra et al. (2018)	2016–2021	6
NP	US‐NP2	mandani2	Cropland	Agriculture	Saliendra et al. (2018)	2016–2021	6
ABS‐UF	US‐ONA	Ufona	Grazing	Shrubland	Gomez‐Casanovas et al. (2020)	2017–2020	4
UMRB	US‐Ro6	rosemountcons	Cropland	Agriculture	Griffis et al. (2005)	2017–2021	5
GB	US‐Rws	arsgreatbasinltar098	Grazing	Shrubland	Flerchinger et al. (2020)	2017–2020	4
SEV	US‐Seg	sevilletagrass	Grazing	Grassland	Anderson‐Teixeira et al. (2011)	2016–2021	6
SEV	US‐Ses	sevilletashrub	Grazing	Shrubland	Anderson‐Teixeira et al. (2011)	2016–2021	6
WGEW	US‐Whs	luckyhills	Grazing	Shrubland	Scott et al. (2015)	2016–2020	5
WGEW	US‐Wkg	kendall	Grazing	Grassland	Scott et al. (2015)	2016–2021	6
NEON‐NGP	US‐xNG	NEON.D09.NOGP.DP1.00033	Grazing	Grassland	Saliendra et al. (2018)	2018–2021	4

Abbreviations: EC, eddy covariance; LTAR, Long‐Term Agroecosystem Research; ROI, region of interest.

**TABLE 2:** Parameter estimates for linear fits of gross primary productivity (GPP) and evapotranspiration (ET) as a function of green chromatic coordinate (G_CC_).

AMF_SITE	PC_SITE	PC_VEG	PRODUCTION	Equation	*R* ^2^
**GPP ∼ G_CC_**					
US‐CF1	cafboydnorthltar01	Agriculture	Cropland	*y* = 106.67*x* + −37.81	0.51
US‐CF2	cafcookeastltar01	Agriculture	Cropland	*y* = 110.08*x* + −35.74	0.65
US‐Jo1	jerbajada	Shrubland	Grazing	*y* = 66.31*x* + −20.58	0.34
US‐Mo1	goodwater	Agriculture	Cropland	*y* = 73.71*x* + −24.01	0.51
US‐Ne1	mead1	Agriculture	Cropland	*y* = 225.05*x* + −76.45	0.74
US‐NP1	mandanh5	Agriculture	Cropland	*y* = 106.8*x* + −35.11	0.79
US‐NP2	mandani2	Agriculture	Cropland	*y* = 93.1*x* + −31.91	0.75
US‐ONA	Ufona	Shrubland	Grazing	*y* = 101.05*x* + −28.9	0.35
US‐Ro6	rosemountcons	Agriculture	Cropland	*y* = 91.35*x* + −30.5	0.59
US‐Rws	arsgreatbasinltar098	Shrubland	Grazing	*y* = 135.46*x* + −43.72	0.68
US‐Seg	sevilletagrass	Grassland	Grazing	*y* = 97.89*x* + −31.51	0.63
US‐Ses	sevilletashrub	Shrubland	Grazing	*y* = 49.81*x* + −16.62	0.42
US‐Whs	luckyhills	Shrubland	Grazing	*y* = 36.54*x* + −11.11	0.29
US‐Wkg	kendall	Grassland	Grazing	*y* = 97.61*x* + −33.79	0.79
US‐xNG	NEON.D09.NOGP.DP1.00033	Grassland	Grazing	*y* = 72.05*x* + −23.15	0.77
**ET ∼ G_CC_**					
US‐CF1	cafboydnorthltar01	Agriculture	Cropland	*y* = 741.68*x* + −247.87	0.48
US‐CF2	cafcookeastltar01	Agriculture	Cropland	*y* = 666.98*x* + −202.11	0.65
US‐Jo1	jerbajada	Shrubland	Grazing	*y* = 1552.51*x* + −487.99	0.26
US‐Mo1	goodwater	Agriculture	Cropland	*y* = 507.23*x* + −142.57	0.35
US‐Ne1	mead1	Agriculture	Cropland	*y* = 1207.68*x* + −385.6	0.65
US‐NP1	mandanh5	Agriculture	Cropland	*y* = 1117.08*x* + −353.37	0.69
US‐NP2	mandani2	Agriculture	Cropland	*y* = 969.56*x* + −313.88	0.63
US‐ONA	ufona	Shrubland	Grazing	*y* = 1474.04*x* + −430.12	0.31
US‐Ro6	rosemountcons	agriculture	Cropland	*y* = 755.78*x* + −239.64	0.51
US‐Rws	arsgreatbasinltar098	Shrubland	Grazing	*y* = 2211.29*x* + −711.16	0.57
US‐Seg	sevilletagrass	Grassland	Grazing	*y* = 1526.3*x* + −486.19	0.38
US‐Ses	sevilletashrub	Shrubland	Grazing	*y* = 1179.57*x* + −392.88	0.29
US‐Whs	luckyhills	Shrubland	Grazing	*y* = 691.56*x* + −199.88	0.18
US‐Wkg	kendall	Grassland	Grazing	*y* = 1323.77*x* + −447.86	0.57
US‐xNG	NEON.D09.NOGP.DP1.00033	Grassland	Grazing	*y* = 1091.63*x* + −349.05	0.72

*Note*: AMF refers to the Ameriflux network site name and PC refers to PhenoCan network site name.

### 2.2 PhenoCam data

We used the 90th percentile G_CC_ timeseries data, capturing the 90th percentile of all G_CC_ values for a given day to avoid spurious values related to changes in illumination (Sonnentag et al., 2012). We used the Phenocamapi R package (Seyednasrollah, 2018) to extract the daily PhenoCam time series for the vegetation region of interest of shrub, grass, or crop (see Table 1 for specific vegetation details). Equation (1) represents how G_CC_ is derived from digital images.

(1) $$\mathrm{Gcc}=\frac{\mathrm{GDN}}{\mathrm{RDN}\;+\;\mathrm{GDN}\;+\;\mathrm{BDN}}$$

where RDN is the red digital number, GDN is the green digital number, and BDN is the blue digital number of the camera's red, green, and blue spectral bands. Years with data gaps greater than seven consecutive days, or if there was no overlap with the flux dataset, were removed.

### 2.3 EC flux tower data

Flux tower data were used to estimate time‐series data for GPP (g C) and ET (mm H_2_O). These data were acquired from AmeriFlux. AmeriFlux FLUXNET (daily timestep) products were used for our analysis (Pastorello et al., 2020). If FLUXNET data products were unavailable for a site, AmeriFlux BASE products (Chu et al., 2023) were used. When BASE products were acquired, data were summarized from the half‐hourly or hourly timestep (whichever was their original form) into daily data. In instances where ET was not included as a variable, we converted latent energy to ET converting latent heat to evaporated water equivalence.

### 2.4 Statistical analysis

We used coefficients of determination (*R* ^2^) to determine the strength and explanatory power of relationships between daily G_CC_ and both daily GPP and daily ET for each site year. To determine differences across vegetation type in the explanatory power of G_CC_ to predict GPP and ET, we performed Kruskal–Wallis and pairwise Wilcoxon signed rank tests. We included Bonferroni adjustment methods for the Wilcoxon rank test to reduce the risk of falsely identifying significant differences due to the increased number of tests performed on repeated measures data. We fit linear models separately for each year and determined differences in slopes across years within each site using Tukey's method for multiple comparisons at significance level *α* = 0.05. To compare the interannual rate of change in GPP and ET as a function of G_CC_ within sites, we used estimated marginal trends from the lstrends() function in the emmeans package (Lenth, 2016). Annual differences in slopes were assessed using pairwise comparisons and were adjusted for multiple comparisons using the Tukey method. To aid in interpretation, we used the cld() function to generate a compact letter display summarizing statistically significant differences. Groups not sharing a letter were statistically different at *α* = 0.05.

## 3 RESULTS

### 3.1 Variability in the explanatory power of G_CC_ in GPP and ET estimates

**FIGURE 2:**
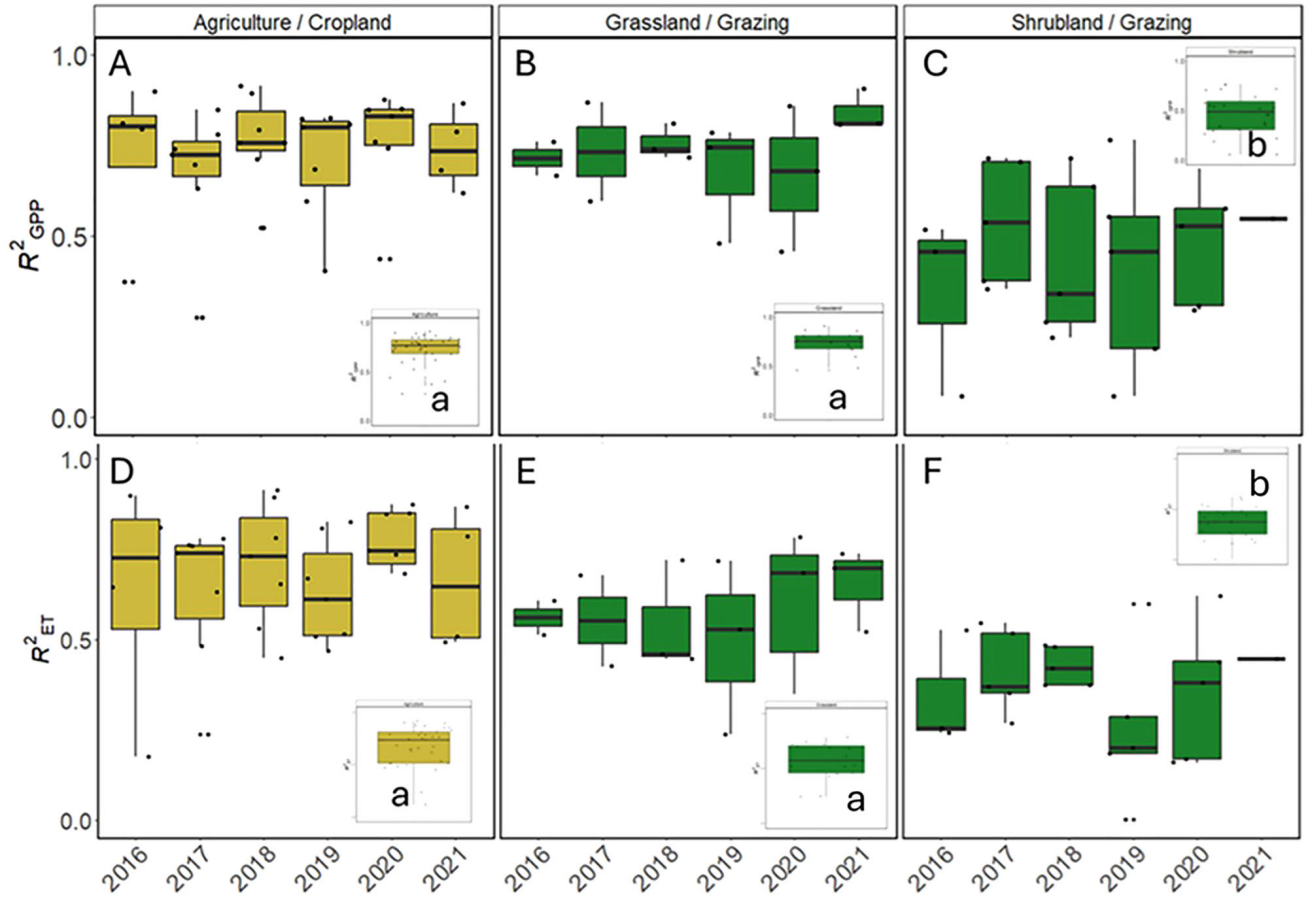
Distribution of *R* ^2^ values associated with the relationships between green chromatic coordinate (G_CC_) and mean daily gross primary productivity (GPP) (A–C) and evapotranspiration (ET) (D–F) across sites within vegetation/production system by year. Colors represent production type (croplands [yellow] and grazing [green]). Panel insets represent range of *R* ^2^ of these relationships across all years within vegetation/production type. Different lowercase letters within insets represent significant differences in explanatory power between vegetation types based on pairwise Wilcox test (α = 0.05).

The explanatory power of G_CC_ is greater for GPP (*R* ^2^ = 0.64) than for ET (*R* ^2^ = 0.54), and less variable in agriculture/cropland, followed by grassland/grazing and shrubland/grazing (Figure 2A–F). The explanatory power of G_CC_ in predicting daily GPP and ET was weaker for shrubland/grazing sites (mean *R* ^2^ = 0.455 and 0.363, respectively) compared to agriculture/cropland (mean *R* ^2^ = 0.727 and 0.662; *p* < 0.001) and grassland/grazing (mean *R* ^2^ = 0.731 and 0.564; *p* < 0.001). The weakest relationships in G_CC_ to GPP occurred in two shrubland/grazing sites: US‐Jo1 in 2016 (*R* ^2^ = 0.06) and US‐Ses in 2020 (*R* ^2^ = 0.26). The weakest relationships in G_CC_ to ET also occurred in US‐Jo1 in 2020 (*R* ^2^ = 0.15) and US‐Ses in 2020 (*R* ^2^ = 0.16). Though these two sites have the weakest single year relationships in G_CC_ to GPP or ET (and the weakest mean *R* ^2^), G_CC_ on average captured 54% and 55% of the variability in GPP and 38% and 45% of the variability in ET for US‐Jo1 and US‐Ses, respectively. Overall, for half of the 76 site‐years evaluated G_CC_ explained ∼70% and ∼50% of the variability in daily GPP and ET, respectively. Further, for 75% of evaluated site‐years, ∼50% (GPP) and ∼40% (ET) of variability was captured in G_CC_.

### 3.2 IAV in the sensitivity of GPP and ET to G_CC_ within sites

**FIGURE 3:**
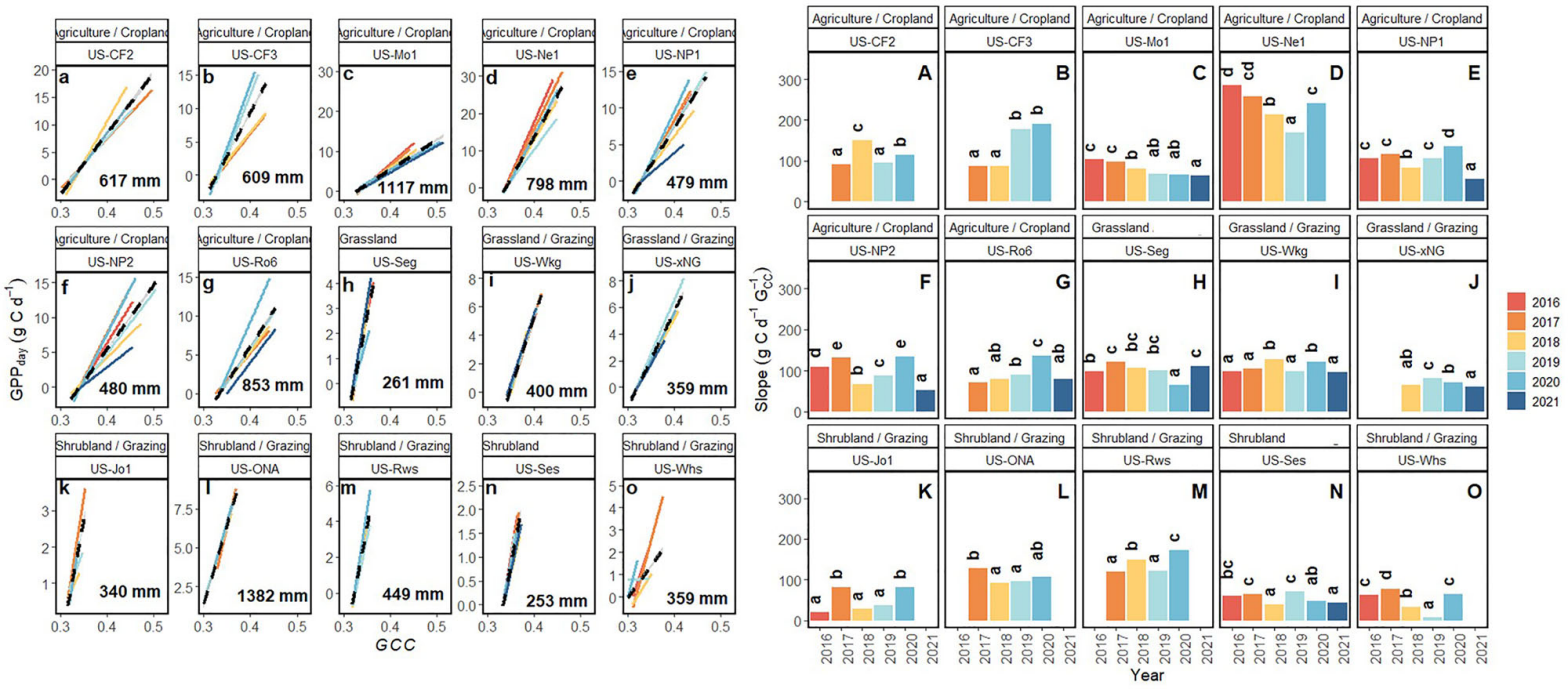
Mean daily gross primary productivity (GPP) as a function of green chromatic coordinate (G_CC_) across years 2016–2021 within each site (a–o). Lines represent linear model fit for each year of data. Black‐hatched lines represent model fit across years for each site (Table 2). Numbers within plots represent the mean annual precipitation. Panels A–O show the slope of the linear regressions represented in a–o demonstrating the sensitivity of GPP to G_CC._ Different lowercase letters represent significant differences in slope across years within a site in panels A–O.

**FIGURE 4:**
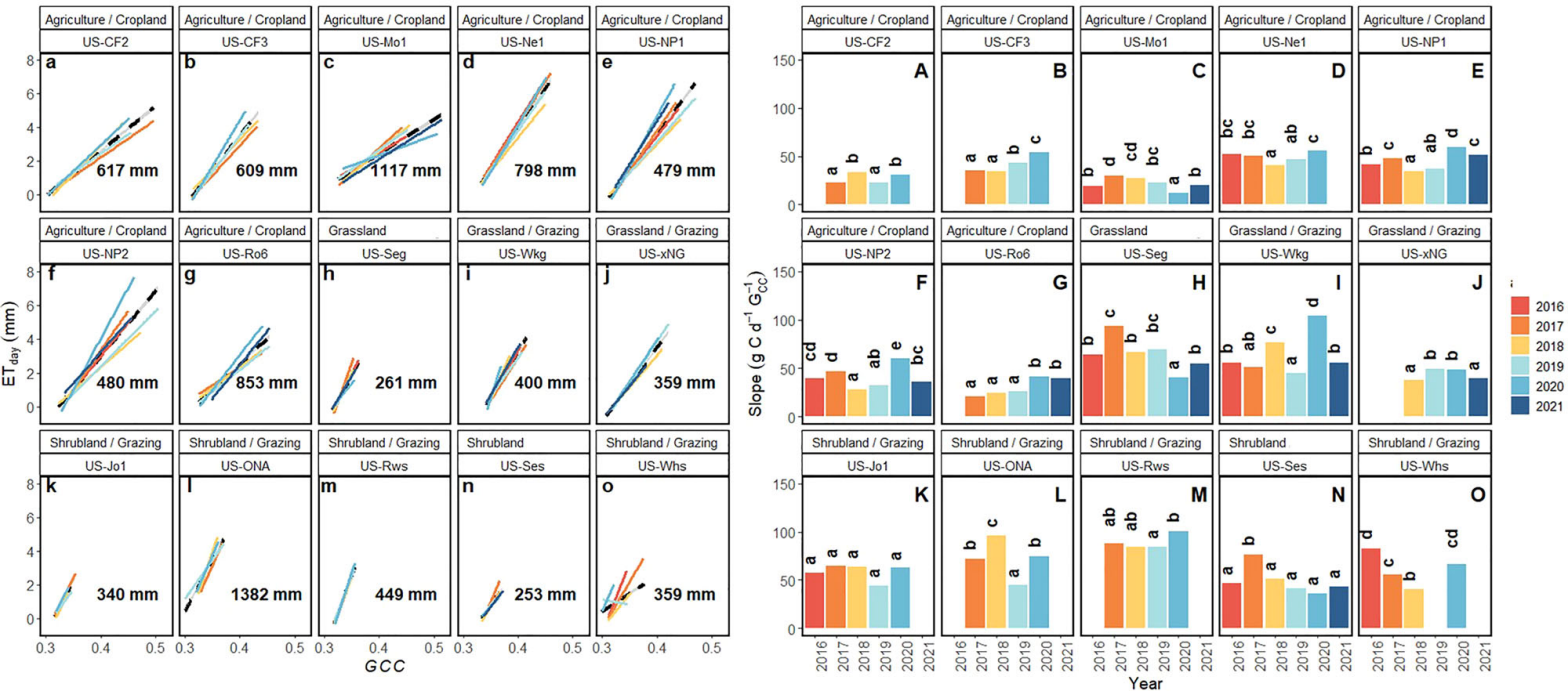
Mean daily ET as a function of green chromatic coordinate (G_CC_) across years 2016–2021 within each site (a–o). Lines represent linear model fit for each year of data. Black‐hatched lines represent model fit across years for each site (Table 2). Numbers within plots represent the mean annual precipitation. Panels A–O show the slope of the linear regressions represented in a–o demonstrating the sensitivity of ET to G_CC._ Different lowercase letters represent significant differences in slope across years within a site in panels A–O.

Linear models between G_CC_ and GPP or ET demonstrate that there is statistically significant IAV between the slope of the relationships within sites. The slopes from the linear models represent the sensitivity of GPP or ET to changes in G_CC_. Simple linear models adequately capture the relationship between GPP or ET and G_CC_ (Figure 2); though both the strength of the overall fit and the rate of change (e.g., sensitivity to changes in G_CC_) varied across years (Figures 3 and 4). The sensitivity of the response of GPP to changes in G_CC_ is more divergent from year to year in agriculture/croplands (Figure 3A–G) compared to both grasslands (Figure 3H–J) and shrublands (Figure 3K–O) observed in pairwise differences across years.

## 4 DISCUSSION

### 4.1 Variability in the strength of G_CC_ to predict GPP and ET within and across agroecosystems

While there is a large range in the explanatory power of G_CC_ to predict GPP or ET across sites and years (*R* ^2^ = 0.06–0.91); G_CC_ explained ∼70% and ∼50% of the variability in daily GPP and ET, respectively, more than half of the time. This suggests that G_CC_ is a reliable metric for informing estimates of both GPP and ET, though consideration of vegetation and production type is required. Shrubland/grazing sites have significantly lower *R* ^2^ compared to both agriculture/cropland and grassland/grazing sites for GPP and ET (Figure 2C,F). Semi‐arid regions, typically consisting of grass and shrub vegetation, contribute ∼50% of the global uncertainty in global carbon flux estimates (Ahlström et al., 2015). Many of the shrubland/grazing sites represented in this study are situated in the western drylands, reinforcing the challenges faced in accurately estimating carbon in these regions. This result further underscores the different environmental drivers of productivity in these diverse agroecosystems; desert shrublands often respond with a lag effect after a precipitation event, benefiting from water reserves from prior events (Sala et al., 2012; Smith et al., 2019).

The weakest relationships between G_CC_ and GPP or ET are observed at the grazed shrublands US‐Jo1 (*R* ^2 ^= 0.06) and US‐Ses (0.26, though grazing in US‐Ses ceased in 1974). Vegetation cover at both sites consists of *Larrea tridentata* (creosote), an evergreen shrub that is remarkably drought tolerant and can maintain photosynthetic activity at extremely high temperatures (Mooney et al., 1978). Alternatively, G_CC_ consistently explained at least 70% of the variability in US‐Rws, located in the Great Basin and dominated by sagebrush. It is surprising that the relationship between G_CC_ and GPP is consistently strong in US‐Rws since it has a semi‐arid climate, much like US‐Jo1 and US‐Ses (Table 1). However, in US‐Rws most precipitation is received during winter, stored, and used in summer, whereas the Chihuahua Desert sites (US‐Jo1 and US‐Ses) have bimodal precipitation regimes, receiving most of the annual precipitation from July to October as the North American monsoon arrives (Munson et al., 2022). The climate of US‐Ses is characterized as cold semi‐arid and US‐Jo1 as cold desert as opposed to semi‐arid steppe of US‐Rws. The IAV in climate plays an important role in structure and function in the region and annual conditions of the greater Chihuahuan Desert are often more characteristic of the semi‐arid steppe (i.e., like that of US‐Rws) (Havstad et al., 2006). It is possible that US‐Jo1 and US‐Ses have effects of interannual carryover of water, resulting in GPP and ET activity of the shrubs remaining relatively constant while green‐up may vary from year to year (Pérez‐Ruiz et al., 2022).

### 4.2 IAV in the sensitivity of GPP and ET to G_CC_ within sites

While we determine that G_CC_ is mostly a reliable predictor of both GPP and ET across all LTAR production and PhenoCam vegetation types (although not all site‐years; see 4.1) examined in this study, the sensitivity of changes in GPP and ET as a function of G_CC_ varies from year to year. Agricultural croplands exhibited more year‐to‐year divergence in the rate of change of mean daily GPP to daily changes in G_CC_, very likely due to crop rotations and timing of harvest. From a modeling perspective, it would be more useful for the sensitivities to align (i.e., converge) regardless of predictive power, as this would allow a static parameter setting from year to year. However, since there is year‐to‐year variability in the rate of GPP increase to increases in G_CC_, a static parameter would not be appropriate. Thus, parameterization would need to be determined based on other environmental drivers influencing rates of change.

Agricultural croplands have more IAV in slope than grazing production types. This result is unsurprising for the PRHRA site (US‐Ne1), which is the only irrigated site in this analysis. Water inputs would result in consistent greenness, while interannual crop rotations would alter GPP each year. It is well understood that irrigation increases productivity, and the added moisture helps to regulate leaf temperature by increasing ET (Kibler et al., 2023), allowing for plants to remain green. For the non‐irrigated sites, the high IAV is likely also due to differences in crop type (i.e., soybean and corn) with the associated different physiologies, planting densities, and productivity. Furthermore, grazing lands (both grassland and shrubland) are not as intensively managed as croplands. While the G_CC_ ‐GPP and G_CC_ ‐ET relationships for these sites may vary year‐to‐year due to changes in environmental conditions such as temperature and rainfall, the relationships for croplands will also be affected by interannual changes in management practices (e.g., fertilizer application, seeding density, or irrigation). It is important to note that the grassland and shrubland sites with the largest variability are highly arid (US‐Ses), where infrequent rain events are likely to have a substantial impact on plant growth. The explanatory power of G_CC_ for ET prediction was less than that of GPP (lower *R* ^2^ for all production/vegetation types; Figure 2), but sensitivities of ET to G_CC_ are less variable than GPP to G_CC_, particularly in grazing shrublands, and points to the high efficiency of water use in water‐limited systems (Huxman et al., 2004). This suggests that when modeling agroecosystem GPP or ET, a static parameter relating G_CC_ to ET may be more reasonable but would not be sufficient for GPP predictions.

Global climate models such as Community Land Model (CLM; Lawrence et al., 2012) or Organizing Carbon and Hydrology in Dynamic Ecosystems (ORCHIDEE, Krinner et al., 2005) model use a broad parameter set that encompasses the dominant vegetation plant functional type (PFT) (e.g., grasses, shrubs, deciduous broadleaf). This approach has many advantages because it simplifies model inputs, distilling a complex parameter into a single term; however, this simplification could result in less accurate projections, as demonstrated in Scheiter et al. (2013). Our results also suggest that using broad PFTs in dynamic vegetation models could be misleading and result in less accurate carbon and water projections. The explanatory power of G_CC_ to both GPP and ET is lower in shrublands (mean *R* ^2^ = 0.46 and 0.36) than grasslands (mean *R* ^2^ = 0.73 and 0.56), in addition to variability in year‐to‐year sensitivity of each to changes in G_CC,_ yet both vegetation types occur within the grazing production classification. This suggests that if a single “Grazing” parameter setting was used to model GPP or ET, models would either over‐ or underestimate carbon and water for the different PhenoCam vegetation types. The coarse resolution of PFT (or, in this case, vegetation and production type) spanning tens of meters to kilometers suggests that more detailed vegetation types should be considered for informing process‐based models (Moon et al., 2022). Ultimately, this can cause PFT‐based models to perform well only in typical homogenous regions (Skidmore et al., 2021) and not be as applicable in heterogenous regions that generally cover the largest proportion of land surface (Smith et al., 2019). Novel approaches for replacing PFT parameter information with Seasonal Characteristics of Vegetation Types and Growth (SCVTG) will further enhance GPP estimation that use remote sensing products due to decreased sensitivity in model outputs when incorporating other input variables (e.g., thermal infrared, a proxy variable for temperature stress) (Zhu et al., 2023).

## 5 CONCLUSION

Ecosystem dynamics are inherently variable from day to day, which is captured with the high temporal fidelity of EC observations but is less apparent from near surface VIs. We hypothesized that the temporal fidelity and high resolution of G_CC_ would be capable of informing estimates of GPP and ET and reveal that G_CC_ accounts for ∼65% of the variability in GPP and ∼55% in ET. While these relationships are variable from year to year and across vegetation or production type, our results indicate that G_CC_ is a strong predictor of GPP in agriculture/cropland, but that in these systems there is a high degree of IAV in the sensitivity of GPP to G_CC_. Though this result is not surprising due to active management of croplands (e.g., crop rotations), it does underscore the need to accurately incorporate growing season metrics as demonstrated in Browning et al. (2021). We find that G_CC_ is a reasonably good predictor of GPP and ET; however, the year‐to‐year differences in sensitivity of GPP and ET to G_CC_ suggest that using G_CC_ as a static parameter in agroecosystem models would be insufficient in predicting GPP or ET, even within a site. Future directions of this work should be geared toward determining the environmental conditions and management decisions impacting the strength in the relationship between G_CC_ and GPP or ET, as well as incorporating an SCVTG (Zhu et al., 2023) variable into near‐surface GPP or ET estimates. Our results demonstrate that PhenoCam derived G_CC_ can be used to predict daily GPP or ET by capturing more rapid changes in vegetation using high temporal frequency, near‐surface imagery, which may be better suited to constrain uncertainty associated with variability in global carbon estimates. The relationship between G_CC_ and GPP was particularly strong in croplands, where frequent management operations make the installation of monitoring equipment difficult. The relative ease of installing PhenoCams makes this a promising approach to monitor crop growth in managed fields as well as plant growth in austere settings.

## AUTHOR CONTRIBUTIONS

**Sander O. Denham**: Conceptualization; data curation; formal analysis; methodology; visualization; writing—original draft. **Dawn M. Browning**: Data curation; supervision; writing—review and editing. **Adam P. Schreiner‐McGraw**: Data curation; writing—review and editing. **Russell L. Scott**: Data curation; writing—review and editing. **Brent Dalzell**: Data curation; writing—review and editing. **Gerald N. Flerchinger**: Data curation; writing—review and editing. **Patrick E. Clark**: Data curation; writing—review and editing. **Sarah Goslee**: Data curation; writing—review and editing. **David L. Hoover**: Data curation; writing—review and editing. **Marcy Litvak**: Data curation. **Marguerite Maritz**: Data curation. **David Huggins**: Data curation; writing—review and editing. **Claire L. Phillips**: Data curation; writing—review and editing. **John Prueger**: Resources. **Joe Alfieri**: Resources; writing—review and editing. **Rosvel Bracho**: Data curation. **Maria Silveira**: Data curation. **Craig W. Whippo**: Data curation; writing—review and editing.

## CONFLICT OF INTEREST STATEMENT

The authors declare no conflict of interest.
